# The Determination, by Chemical Reagents, of the Identity or Non-Identity of Specimens of Writing Ink upon Paper

**Published:** 1859-02

**Authors:** 


ART. III.—*The Determination, by Chemical Reägents, of the Identity or Non-identity of Specimens of Writing Ink upon Paper.* By the EDITOR.

There is no subject in scientific jurisprudence, which has bestowed upon it an attention less commensurate with its importance, than the chemical examination of inks, as presented to us in ordinary specimens of writing. One can easily see the numerous applications which could be made, of an exact process, by which the chemist could assert that certain characters were written with the same, or with different inks; but strange to say, there are few books upon chemistry which pretend to give the composition, even, of the common varieties of ink, and nothing written which treats of the comparison of different specimens. It would, of course, be impossible to give the exact components of many celebrated inks, as this is a secret of the manufacturers, and a problem which has baffled many excellent chemists.

The following case is one of extreme interest, as the guilt or innocence of an individual was almost entirely dependent upon the fact of the identity or non-identity of two marks upon the same bit of paper. I will be unnecessary, for my present purpose, to give a full account of the case, and will be sufficient to note its more important features, an account of which was kindly furnished me by Mr. Albert Sawin, of this city, the able counsel for the defendant.

The case was tried in the last Criminal Term of the Superior Court, Judge Masten presiding.

"The defendant was indicted for forgery, and the alleged forgery consisted in making a cross (×), as the mark of one Joseph Umbleby, after the words "Joseph Umbleby, his mark," upon three notes of $322 each.

The people proved by four witnesses, Joseph, the father, and his three sons, in substance, that on October 15th, 1658, the father, Joseph Umbleby, although requested by his sons to sign the note as their security, and after at first having agreed to do so, finally refused, and did not sign, and that this refusal was made known to the defendant, who agreed to close the bargain with the sons without the father's signature, and that the notes were delivered to the defendant without the (×).

These witnesses were apparently fair and honest men, and quite intelligent. No impeachment of their general character was attempted.

To overcome this proof, apparently so impregnable, the defendant relied upon a variety of circumstances, but the counsel for him stood upon this position:

The body of the notes, and the words "Joseph Umbleby, his mark," were written October 14th, 1856, at the father's house in Hamburgh, (a country town near Buffalo,) by one of his sons, the only ink in the house, or that had been for months, being contained in a little sixpenny bottle, which was also used as ink-stand. *The ink of the mark was the same as that of other parts of the note.*

The defendant's counsel concluded if it was a different ink, his client was guilty. The people's case was such, "that if it could be proven that all parts of the note were written with the same ink, from the same inkstand, the defendant was innocent."

The defendant's counsel applied to me some time ago, to test these signatures, and ascertain, if possible, the fact of the identity or the non-identity of the ink which composed the signature, "Joseph Umbleby" and the mark. I was applied to on account of the absence of Prof. Hadley, and the necessity of an immediate establishment of that point. As I had never undertaken anything of the kind before, and was not able to find any definite instructions in the books, I hesitated to assume the responsibility, and was not at all sanguine of the benefit which would be derived from my examination; nevertheless, I consented to undertake to make something out of it. After instituting a variety of experiments, I found, that on the application of dilute sulphuric acid to different specimens of ink, taken at random from old letters, etc., a color was produced, which was of a different shade in every specimen which was examined. This experiment, being repeatedly performed, seemed to establish a basis on which I could form an opinion of the inks in question, from such an application. The following is substantially the testimony which was given in relation to this point at the first trial, when the jury failed to agree:

In addition to the preparatory examinations which were made, with regard to the color produced by the application of dilute sulphuric acid to different specimens of ink, ten observations were made on specimens taken from the backs of old letters, dated at about the time that the notes were made. In these observations, two small bits of the paper, on which was a portion of writing, were cut out with the scissors, placed on a slip of glass, dilute sulphuric acid was applied to them simultaneously, and they were submitted together to a magnifying power of sixty diameters, so that they could both be examined at the same time, without moving the specimen. The examinations were made with the lowest power of Nachet's large instruments, and by reflected light. The results of these ten comparisons were then recorded, and it was found that no two presented precisely the same shade of color. In the first observation, a very marked difference in shade was observed after the the application of the acid, though before its application, it would have been utterly impossible to have remarked any difference between the specimens. The colors produced were different shades of a light blue.

In the second observation, the result was the same; a light blue color was produced by the acid, but the shades presented a marked difference.

In the third observation, the result was highly satisfactory, a blue being produced in one specimen, and dirty brown in the other. The latter was from the back of a letter from a country town.

In the fourth observation, there was a very marked difference in shade, both being of a blue color.

In the fifth observation, the difference in color was very marked indeed; before adding the acid, the two specimens could not be distinguished from one-another; but after its application, a blue color was produced in one instance, and a pink in the other.

In the sixth observation, the same results followed; one specimen was rendered blue and the other pink, by the application of the acid.

In the seventh observation, the difference was very marked; one specimen was blue, and the other green.

In the eighth observation, one specimen was blue, and the other pink.

In the ninth observation, one specimen was blue, and the other brown; the latter was from a country letter.

In the tenth observation, the difference in shade was not great, but was perfectly apparent.

From the examinations which I had made in advance of these ten observations, I was convinced that it was possible to distinguish between two specimens of ink upon paper, by the application of an acid; and for my reagent, I chose the dilute sulphuric acid, though I was afterwards informed by Prof. Hadley that the muriatic would have been preferable, not because the immediate effect would have been different, but because the specimens could have been preserved without rotting the paper, as would be the case when the sulphuric acid were used. The importance of the scientific testimony in this case, however, and the necessity, not of discriminating between two varieties of ink, but of showing that two marks were made with the same ink, made it advisable to establish for my testimony the basis of a few recorded observations.

It will be seen by these observations, that no two inks presented the same shade after the application of the acid: four of the specimens were blue, but of different shades: three were pink, with a marked difference in shade; two were brown, of a different shade; one was green. It made no difference whether the mark was heavy or light; the same *shade* was produced by the acid in the same specimen, and a different shade in different specimens. Care was taken that two specimens should be in the field of vision at the same time, and they were placed directly in apposition to each other. All these observations were confirmed by Prof. Flint, who was present at the examinations.

Having established the above points, attention was now directed to the notes; the comparison of the signature "Joseph Umbleby," the mark which followed it, the body of the notes, and the signatures of the two sons.

The first examination was of the signature "Joseph Umbleby," and the mark. Bits of paper, bearing the ink of the marks and signature were cut out of the note, placed in apposition upon a slip of glass, treated with dilute sulphuric acid, and submitted to a magnifying power of sixty diameters. They showed *exactly the same shade of a dirty brown, with no possible difference.* They were compared carefully and repeatedly by myself, and the fact was confirmed by Prof. Flint.

An examination was then made of other pieces cut from the signature and the mark. They were treated in the same way, and the experiment was followed by the same results.

A third observation was made, confirming the two foregoing.

Comparison was then made between the signature Joseph Umbleby, and the signature of the two sons, which were acknowledged to have been written with different inks, a fact which was apparent before the application of the acids. Here was found a marked difference, one being brown, as was said before, and the others blue.

From the facts above stated, I testified that, in my opinion, the mark in question was made with the same ink as the signature "Joseph Umbleby."

In the first trial, the testimony was precisely the same; but Prof. Hadley not being in the city, and it was not, as now, confirmed by a professed chemist. Before the last trial, however, I saw Prof. Hadley, gave him an account of my experiments, and he consented to repeat them. Though muriatic acid would have been preferable, for reasons which have been stated, Prof. Hadley used the sulphuric; as that was the reägent used in the previous experiments, and as it was not essential that the pieces of paper should be preserved. He made a few experiments upon different inks, which were entirely confirmatory of my own, and by pasting the specimens, treated in this way, opposite to each other on a blank sheet of paper, he enabled the jury to see, at a glance, the difference in shade between the different varieties of ink, and the identity in shade between the mark and the signature.

Prof. Hadley was of the opinion, that the mark and signature were made with the same ink, and probably at the same time; he stated that the ink used was of an old variety, and such as is sold in large quantities in country towns. He added, upon cross-examination, that he thought it possible to discriminate between two specimens of ink, and to determine, almost, if not quite, positively, that two marks were made with the same ink, and at the same time; that ink was subject to chemical changes, and that these changes would not be the same, even in two specimens of the same ink, unless they were exposed to precisely the same influences, were present in the same quantities, and were used equally.

Mr. C. R. Walker, who is engaged in the establishment of Mr. A. I. Mathews, was called to the stand, and testified that he had tested the signature and body of the note and mark with the cyanide of potassa, and that they indicated iron in about the same proportion. He used acids upon them with the same effect. He examined scrapings of the ink with the microscope, and the granules presented the same appearance. He should have no hesitation in saying that they were written with the same ink.

The importance of the scientific testimony in this case was strongly urged upon the jury by the able counsel for the defendant, and after twenty minutes deliberation, they brought in a verdict of acquittal.

This case is instructive to us as scientific men, as we are liable to be applied to at any time, to examine signatures, and give an opinion in regard to the identity or non-identity of inks. I have, therefore, given the full account of my experiments, endeavoring to show that it is no difficult matter to make such an investigation, and that the results will generally be satisfactory. The precise chemical reäctions which are induced by the application of an acid, I do not pretend to explain; but that a different color is produced by the application of acids to different specimens of writing, seems to me to be pretty well established. Did we know the exact chemical composition of the inks now in use, we would be able to make our experiments much more intelligently; but that is a secret of the manufacturers, and some of the popular varieties, Arnold's writing fluid, for example, have defied the analytical skill of the most experienced chemists. Of course such examinations should, if possible, be always made by an educated chemist; but there might be circumstances, like those under which the writer was placed, where it would be necessary for a physician, not particularly skilled in chemistry, to give an opinion in such a case. For the benefit of those to whom this may happen, I will give the following conclusions, which I have derived from my experience in this single case:

Any acid of a proper strength may be used, but the muriatic is preferable, as it will not rot the paper.

The acid, applied to the specimens under examination, will produce a change in color. This will be generally blue, sometimes pink, sometimes brown, and sometimes, though rarely, green; occasionally, the mark is almost obliterated.

The color thus produced may be examined with a low magnifying power, by reflected light, or with the naked eye, which latter will commonly answer all purposes, and a difference in shade will be observed in different inks.

